# Volumetric MRI study of the brain in patients with neurocysticercosis and mesial temporal lobe epilepsy

**DOI:** 10.1002/epd2.70115

**Published:** 2025-11-14

**Authors:** Jaisa Quedi Araújo, Bruna da Silveira Arruda, Sandra Polita, Maurício Anés, Thiago Junqueira Ribeiro de Rezende, Matheus de Lima Ruffini, Adolfo Moraes de Souza, Jordana Soares Chaves, Fabiano Reis, Marino Muxfeldt Bianchin, Juliana Ávila Duarte

**Affiliations:** ^1^ Hospital de Clínicas de Porto Alegre Universidade Federal do Rio Grande do Sul Porto Alegre Brazil; ^2^ Faculdade de Ciências Médicas Universidade Estadual de Campinas Campinas Brazil

**Keywords:** magnetic resonance imaging, mesial temporal lobe epilepsy, neurocysticercosis, volumetric cerebral

## Abstract

**Objective:**

Neurocysticercosis (NCC) is a common parasitic infection of the central nervous system and a known cause of focal epilepsy. Its potential role in triggering or contributing to mesial temporal lobe epilepsy with hippocampal sclerosis (MTLE‐HS) is suggested, but the impact on brain volumetry remains unclear. This study aimed to assess volumetric differences in the brain, with a particular focus on the hippocampus and temporal lobes, in patients with NCC, MTLE, MTLE‐HS, and their combinations.

**Methods:**

This was an observational, cross‐sectional, retrospective, single‐center exploratory study including five groups: healthy controls; isolated MTLE; isolated NCC; MTLE‐HS; and MTLE‐HS combined with NCC (MTLE‐HS plus NCC). All participants had at least one magnetic resonance imaging (MRI) examination suitable for volumetric post‐processing. Volumetric analysis was performed on cerebral cortex regions (frontal, parietal, temporal, and occipital lobes), cerebellum, total and white matter limbic system, amygdala, hippocampus, and thalamus using the T1 MultiAtlas Segmentation tool (Brain GPS©). We also assessed sensitivity by estimating the minimum detectable difference from adjusted models and performed an exploratory analysis of NCC lesion topography with Welch *t*‐tests on lobar/subcortical volumes.

**Results:**

After adjusting for age and sex, no significant group differences were found in hippocampal or temporal lobe volumetrics (all *p* > .10). Effect sizes were small (partial η^2^ ≤ .10), and MANOVA confirmed the absence of a global effect (Pillai's Trace = .114, *p* = .623). Adjusted means showed only modest variation with overlapping confidence intervals, indicating that differences likely reflect individual variability rather than systematic group effects.

**Significance:**

The findings indicate that NCC, whether isolated or combined with MTLE‐HS, does not cause significant alterations in brain volume. This suggests the absence of an additive or synergistic effect of NCC on brain morphostructural changes in patients with MTLE‐HS. These results contribute to understanding the pathophysiology of epilepsy related to NCC and hippocampal sclerosis, supporting that volumetric brain changes may not underlie their association.

**Key points**

- No significant brain volume differences were found among patients with NCC, MTLE, MTLE‐HS, or their combinations.
- NCC, whether isolated or combined with MTLE‐HS, does not produce measurable brain volumetric changes on MRI.
- There is no additive or synergistic effect of NCC on brain morphostructural changes in patients with MTLE‐HS.
- Automated MRI volumetric analysis is feasible and reliable in these patient groups.
- Findings highlight the need for larger studies to confirm these results.

## 1 INTRODUCTION

Neurocysticercosis (NCC) is one of the most common parasitic diseases of the central nervous system and a major cause of focal epilepsy worldwide, affecting millions of patients.^1^, ^2^ It is an infection caused by the cystic form of the pig's tapeworm, Taenia solium, a helminth of the flatworm type, which belongs to the Cestoda class.^3^

Epidemiological studies suggest that neurocysticercosis is a major cause of symptomatic epilepsy in developing countries.^4^, ^5^ The association between NCC and mesial temporal lobe epilepsy (MTLE) is discussed by some authors. NCC appears to emerge as a causative or contributing agent to the development of MTLE in some patients.^6^ This association is important because it can have an impact on the evaluation and treatment of a considerable proportion of patients with epilepsy.^7^

A pathogenic relationship between neurocysticercosis and mesial temporal lobe epilepsy associated with hippocampal sclerosis (MTLE‐HS) has been proposed, with the former representing an initial precipitating injury to the hippocampus, eventually leading to MTLE‐HS.^8^ However, the mechanisms behind this association are not yet fully understood.

The radiological appearance of neurocysticercosis varies depending on the stage of the disease and determines the appropriate treatment.^9^, ^10^ Where this pathology is endemic, chronic calcified neurocysticercosis (cNCC) can be seen in patients with mesial temporal lobe epilepsy associated with hippocampal sclerosis (ELTM‐HS).^11^, ^12^, ^13^, ^14^

Neuroimaging is very important to find out about this possible relationship between neurocysticercosis and MTLE‐HS.^9^ Our study aims to evaluate the volumetry of specific brain structures—particularly the hippocampus and temporal lobes—in order to investigate whether volumetric alterations are associated with the presence of epilepsy (MTLE), hippocampal sclerosis (MTLE‐HS), and neurocysticercosis (NCC), in both isolation and combination. Furthermore, volumetric data from these groups were compared with those of control patients without comorbidities, providing a normative reference to enhance the accuracy and clinical interpretability of the findings.

## 2 METHODS

This observational, cross‐sectional, retrospective, single‐center, exploratory study followed the STROBE statement. Participants were distributed into five groups: controls; isolated mesial temporal lobe epilepsy (MTLE); isolated neurocysticercosis (NCC); mesial temporal lobe epilepsy with hippocampal sclerosis (MTLE‐HS); and mesial temporal lobe epilepsy with hippocampal sclerosis and neurocysticercosis (MTLE‐HS plus NCC). Additionally, patients with hippocampal sclerosis were subdivided according to the lateralization of their pathological manifestation (right or left). The research recruited patients from Hospital de Clínicas de Porto Alegre (HCPA), a tertiary university hospital in southern Brazil. All participants, both those with pathologies and controls, used the same MRI machines from the institution. Potential participants were identified through a search of MRI reports containing key terms (“neurocysticercosis”, “cysticercus”, “calcifications”, “epilepsy”, and “hippocampal sclerosis”) on the Impacs® platform, the picture archiving and communication system (PACS) used at HCPA. The search was carried out from March to November 2020, covering reports of MRI scans performed between January 2013 and March 2020. All reports that included at least one of the key terms above, in any field, were selected for further screening based on eligibility criteria, including data from the full report, the image sequences available, and the electronic patient records.

### 2.1 Eligibility criteria

For patients with epilepsy, the diagnoses of MTLE and MTLE‐HS were established by experienced epileptologists based on unequivocal clinical features of epileptic seizures, supported by electroencephalographic and neuroimaging findings. The diagnosis of NCC was determined by neurologists or radiologists according to clinical and neuroimaging criteria. Control subjects had no history of neurological disease and underwent brain MRI for unrelated reasons, with reports classified as normal.

All participants were required to have at least one MRI performed at the Radiology and Diagnostic Imaging Service of HCPA, including a volumetric T1 sequence with a minimum of 170 slices.

### 2.2 Exclusion criteria

Patients were excluded if they presented clinical histories suggestive of non‐epileptic paroxysmal disorders (e.g., syncope, psychogenic seizures, and transient ischemic attack), other neurological conditions, or incidental MRI abnormalities (except mild microangiopathy—Fazekas I). Individuals with a history of neurosurgical interventions such as amygdalo‐hippocampectomy, temporal lobectomy, or other procedures performed before the available MRI were also excluded. For controls, exclusion criteria included any incidental brain MRI findings beyond mild microangiopathy (Fazekas I).

To minimize selection bias and enhance external validity, the eligibility criteria were deliberately broad. Clinical and demographic data collected in 2019 included sex, age at MRI, age at epilepsy onset, history of febrile seizures, family history of epilepsy, seizure‐free status, and antiepileptic drug use at the time of MRI. Patients with missing data were excluded from the analyses.

### 2.3 MRI acquisition

MRI scans included in this study had been previously acquired as part of each patient's routine clinical care using scanners located at HCPA's Radiology and Diagnostic Imaging Service, according to a standardized protocol. Only scans including a volumetric T1 sequence with at least 170 images were included to satisfy the minimum requirements for volumetric analyses. Images were acquired using two MRI scanners from Philips: one 1.5 Tesla scanner, model Achieva, software version 2.6.3, with an 8‐channel head coil, and one 3.0 Tesla scanner, model Ingenia, software version 5.4.1, with a 15‐channel head coil. For brain structural assessment, a turbo field echo (TFE) acquisition with inverse preparation pulse was used. In the 1.5 Tesla scanner, images were acquired in the sagittal plane with the following parameters: TR = 7.0 ms, TE = 3.2 ms, TI = 840 ms, flip angle = 8°, number of signal averages/acquisitions = 1, voxel size = 1 mm^3^. In the 3.0 Tesla scanner, images were acquired in the axial plane with the following parameters: TR = 7.9 ms, TE = 3.5 ms, TI = 950 ms, flip angle = 8°, number of signal averages/acquisitions = 1, voxel size = 1 mm^3^.

### 2.4 MRI post‐processing

Brain volumetric analysis was performed using the T1 MultiAtlas Segmentation tool (https://braingps.mricloud.org/t1prep), part of Brain GPS©, a free service for processing T1 images and converting them into 286 segmented structures based on the brain ontology of the JHU multi‐atlas inventories, as described by Chupin et al.^15^ We selected the most affected structures in the pathologies under analysis.^16^

This neuroinformatics pipeline uses an atlas‐based approach (Figure S1), “deforming” a set of atlases to “match” the individual brain under analysis and, subsequently, applying the deformations to a corresponding set of segmentations. It then fuses probability across the set of segmentations, resulting in the segmentation of individual brain data. Ultimately, volumes of all brain structures are provided.

In our research, we separated for analysis the volume provided by the program of the following structures: cerebral cortex, frontal lobe, parietal lobe, temporal lobe, occipital lobe, cerebellum, limbic system, limbic system WM (white matter), amygdala, hippocampus, thalamus.

Demographic and clinical data were collected from patient records by a trained physician (BSA), using specific forms, and were entered anonymously into an electronic datasheet using Excel (Microsoft Corporation, USA). All appropriate measures were taken to prevent confidentiality breaches or data loss and to ensure data quality.

### 2.5 Statistical analyses

Continuous variables were expressed as mean and standard deviation (SD) or median and interquartile range (IQR), depending on their parametricity, which was assessed by means of the Shapiro–Wilk test. Categorical variables were expressed by means of absolute and relative (%) frequencies.

Group comparisons of hippocampal and temporal lobe volumes were performed using analysis of covariance (ANCOVA). For each metric (right, left, and total hippocampal volumes; right, left, and total temporal lobe volumes), we fitted linear models with group as the main predictor and age and sex as covariates. The Control group (C) was excluded from inferential analyses, but its mean values were retained as a normative reference for descriptive purposes. Additionally, a multivariate analysis of variance (MANOVA) with Pillai's Trace test was conducted for the three hippocampal measures simultaneously, adjusting for the same covariates. Adjusted marginal means (least‐squares means) were estimated at the mean age of the sample and with sex balanced at 50/50 to facilitate clinical interpretation. All analyses were performed using the RStudio software (R version 4.4.1, RStudio version 2024.04.2).

To estimate the smallest globally detectable volumetric reduction among EP, EPESC, EPNC, and NC (with group C excluded from inferential tests), we performed a one‐way ANOVA (*k* = 4) followed by an ANCOVA adjusting for age and sex. For each metric—Right Hippocampus, Left Hippocampus, and Total Hippocampus (Right + Left)—we obtained the residual standard deviation from the adjusted model (volume ~ age + sex). From the critical effect size that ensures 80% power for the global test (Cohen's f* ≈ .429; α = .05), we converted the effect into a minimum detectable difference (Δ_min) under the most favorable partition of the test (maximizing *p*(1 − *p*), with p(1 − *p*) ≈ .249 given the sample distribution EP = 17, EPESC = 20, EPNC = 11, NC = 19). For clinical interpretation, Δ_min was also expressed as a percentage of “normal,” where “normal” explicitly corresponds to the mean of the Control group (C) for the given metric; note that C does not enter the inferential tests and serves only as a normative anchor for the percentage scale. An identical procedure was applied to the Right, Left, and Total Temporal Lobe volumes, ensuring methodological consistency across both hippocampal and temporal structures.

Finally, to assess whether NCC lesion location or burden relates to regional brain volumes, we summed right + left lobar/subcortical volumes and expressed lobar volumes as a fraction of total cortex to mitigate head‐size effects; groups with vs. without a lesion in a given lobe were compared with two‐sided Welch *t*‐tests on absolute and normalized measures, and hippocampal/amygdalar volumes were examined when temporal involvement was present.

### 2.6 Ethics

This study was approved by the Research Ethics Committee at the School of Medicine, Federal University of Rio Grande do Sul (CAAE 70143417.9.0000.5327), and was carried out in accordance with Brazilian National Health Council‘s Resolution number 196/1996, version 2012.

## 3 RESULTS

**FIGURE 1:**
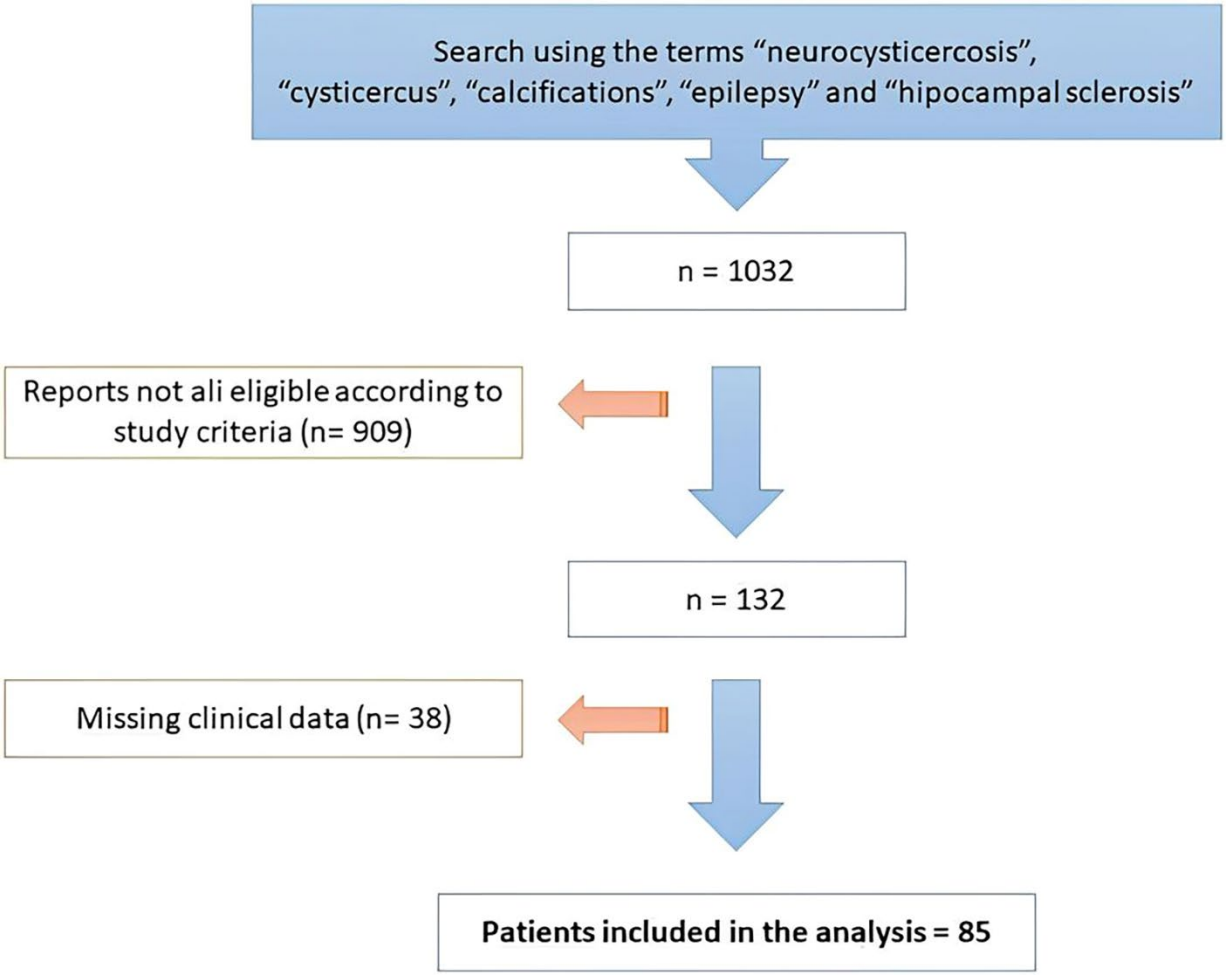
Diagram showing sample selection strategy.

Following the search for key terms on the Impacs system, 1032 reports of potentially eligible MRI scans were retrieved. Of these, only 123 individuals met all the eligibility criteria and had their medical records reviewed for clinical data, but important data were missing for 38 individuals; therefore, only 85 individuals were included in the analyses (Figure 1).

The distribution of subjects across study groups, along with their demographic and clinical features, is summarized in Table 1.

**TABLE 1:** Study group, demographic and clinical characteristics.

Group	NCC (*n* = 19)^a^	MTLE (*n* = 17)	MTLE‐HS (*n* = 20)	MTLE‐HS + NCC (*n* = 11)	Control (*n* = 18)	*p*‐Value
Male—*n* (%)	7 (36.8%)	5 (29.4%)	9 (45.0%)	3 (27.3%)	10 (55.6%)	.4571
Female—*n* (%)	12 (63.2%)	12 (70.6%)	11 (55.0%)	8 (72.7%)	8 (44.4%)	
Age—*n* (%)	47.0 (14.6)	44.8 (13.2)	46.7 (14.3)	55.5 (11.9)	41.4 (16.6)	.161
Age at first seizure, in years —median (IQR)	33.5 (31.5)	15 (10)	10 (8.5)	10 (6.5)	NA	<.001
Time since first seizure, in years—mean (SD)	18 (14.98)	24 (14.12)	35.85 (15.17)	48.09 (13.29)	NA	<.001
Number of antiepileptic drugs at last follow‐up—median (IQR)	2 (1)	1 (1)	2 (1)	3 (1)	NA	.0494

Abbreviations: HS, hippocampal sclerosis; MTLE, mesial temporal lobe epilepsy; NA, not applicable; NCC, neurocysticercosis; SD, standard deviation.

^a^ Eight patients had epilepsies not classified as MTLE.

**FIGURE 2:**
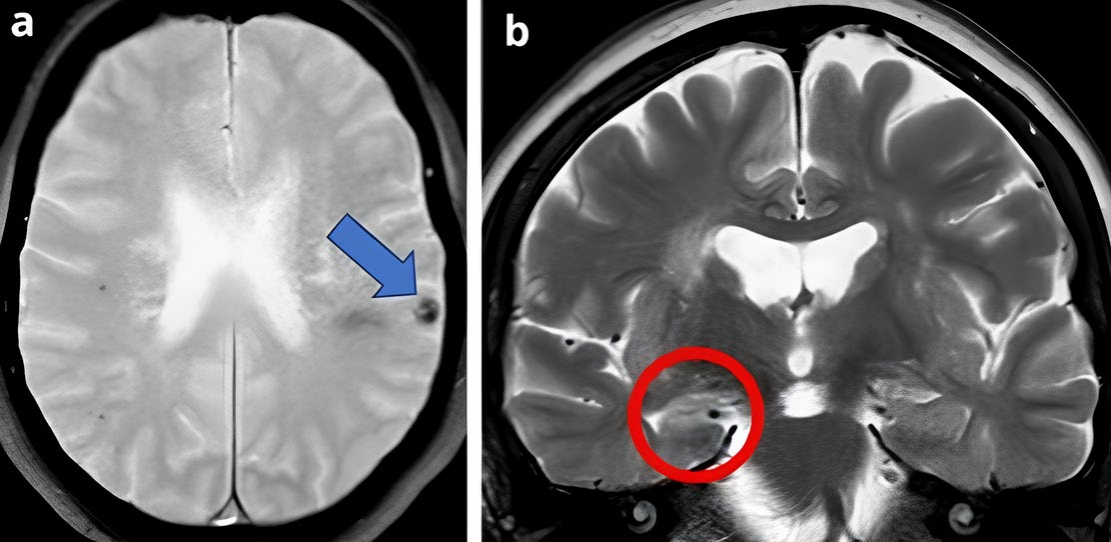
Magnetic resonance images in a patient with MTLE‐HS plus NCC. (A) Axial, T2‐gradient‐echo image showing a hypointense nodular lesion in the left parietal cortex (blue arrow), consistent with calcified neurocysticercosis. (B) Coronal, T2‐weighted image showing increased signal intensity and atrophy of the right hippocampus (red circle), compatible with hippocampal sclerosis.

As an example, Figure 2 shows the magnetic resonance imaging (MRI) of a patient with neurocysticercosis and mesial temporal lobe epilepsy with right‐sided hippocampal sclerosis.

**FIGURE 3:**
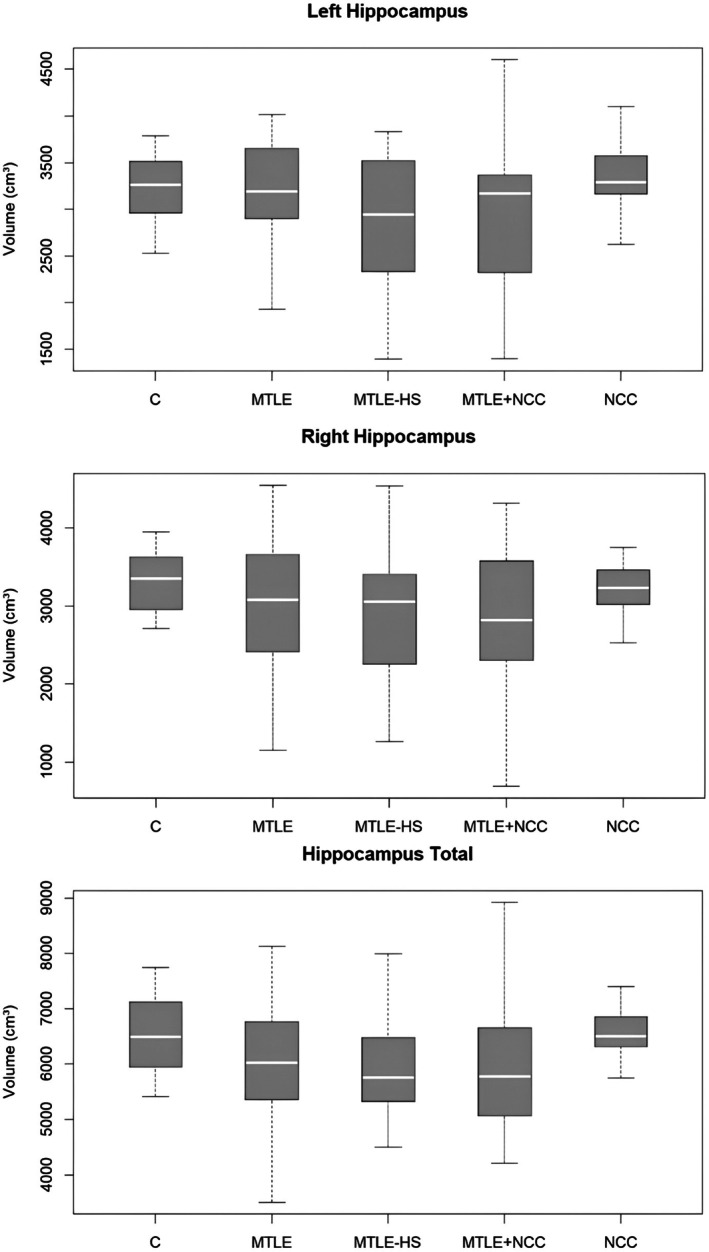
Hippocampal volumes across study groups: Left, right, and total measurements. C, control; HS, hippocampal sclerosis; MTLE, mesial temporal lobe epilepsy; NCC, neurocysticercosis.

**FIGURE 4:**
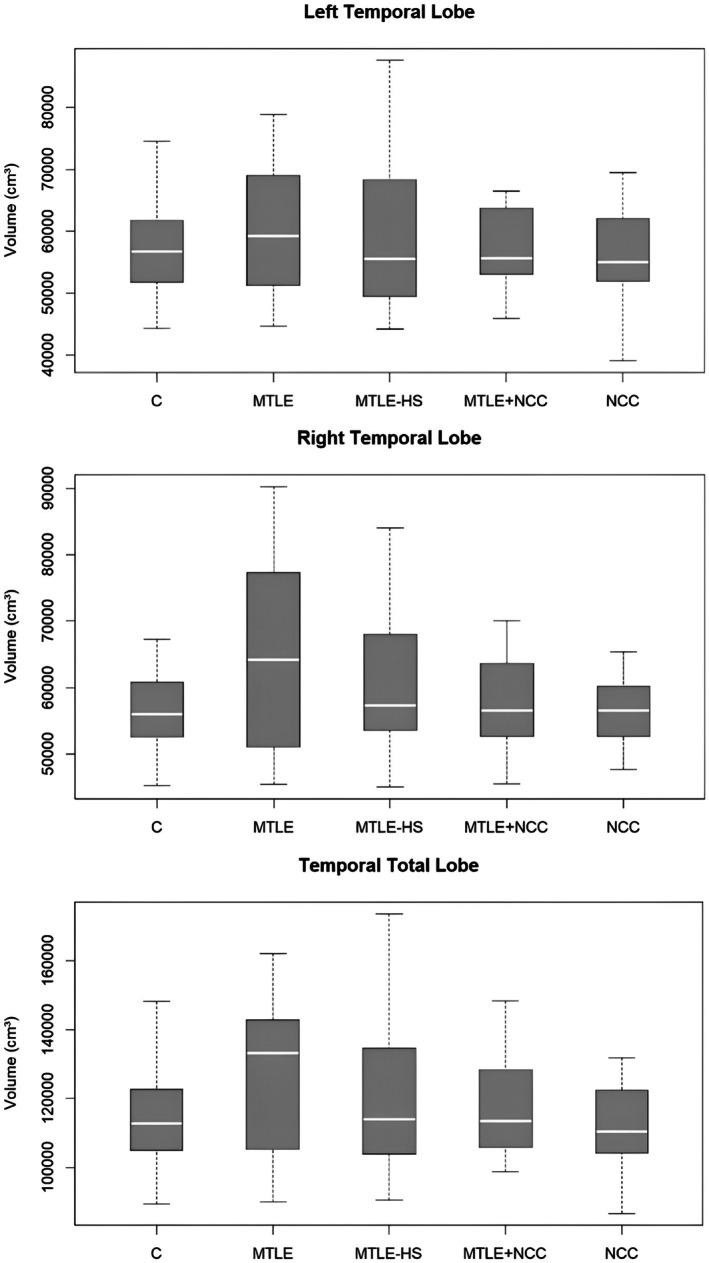
Temporal lobe volumes across study groups: Left, right, and total measurements. C, control; HS, hippocampal sclerosis; MTLE, mesial temporal lobe epilepsy; NCC, neurocysticercosis.

For both hippocampal (Figure 3) and temporal lobe volumes (Figure 4), the boxplots show similar medians and a wide overlap of interquartile ranges across the groups. While the MTLE‐HS and MTLE+NCC groups display a slight trend toward smaller volumes, this difference is neither consistent nor statistically significant, suggesting that the findings reflect individual variability rather than a systematic group effect. Boxplots for the remaining brain structures assessed are provided in the Figures S2–S9.

After adjustment for age and sex, no statistically significant differences were observed among NCC, MTLE, MTLE‐HS, and MTLE + NCC groups for any of the hippocampal or temporal lobe volumetric metrics (all *p* > 0.10). Effect sizes were small (partial η^2^ ≤ 0.10), and the MANOVA confirmed the absence of a significant global group effect across hippocampal measures (Pillai's Trace = 0.114, *p* = 0.623). The adjusted means indicated only modest volumetric variation across groups, with broad overlap of confidence intervals, suggesting that the observed differences reflect individual variability rather than systematic group effects.

In relation to the minimum detectable volumetric reduction, the age‐ and sex‐adjusted model indicated thresholds of Δ_min ≈ 662.5 mm^3^ for the Right Hippocampus (≈20.0% of the Control group average), Δ_min ≈ 732.6 mm^3^ for the Left (≈22.7%), and Δ_min ≈ 1095.5 mm^3^ for the Total Hippocampus (≈16.8%). For the temporal lobe, the corresponding thresholds were Δ_min ≈ 10157.4 mm^3^ for the Right (≈18.0% of the control reference), Δ_min ≈ 8633.7 mm^3^ for the Left (≈15.1%), and Δ_min ≈ 16771.6 mm^3^ for the Total Temporal (≈14.8%). Overall, the total volumes (hippocampal and temporal) consistently provided the most favorable sensitivity, requiring the smallest percentage reduction relative to healthy controls to be detectable.

Regarding the relationship between NCC lesion location and regional brain volumes, the temporal lobe showed a nonsignificant trend toward a smaller normalized fraction in participants with lesions (0.2358 vs. 0.2428; Δ = −0.0070; *p* = 0.0556). No significant differences were observed for the hippocampus, amygdala, parietal, or occipital lobes. Overall, the only suggestive finding was a possible reduction in temporal lobe volume with lesions, which did not reach statistical significance and should be interpreted with caution; confirmation will require larger and better‐powered samples.

## 4 DISCUSSION

The comparison of volumetric brain parameters in conditions such as MTLE, MTLE‐HS, and neurocysticercosis, alone or in combination, can provide information on the differential contribution of these conditions to brain diseases.^17^, ^18^, ^19^, ^20^ In this study, we examined whether the analysis of the volumetry of brain structures showed changes in patients with the pathologies described above in comparison with asymptomatic control patients, or whether they could support the hypothesis that the combinations of the above conditions are worse than the conditions alone. However, our data did not demonstrate consistent differences between the study groups.

We were able to assess that patients with pathologies alone or in combination did not show a significant difference from the volumetric brain evaluation, suggesting that there is no additive action of these pathologies in causing brain morphostructural changes. Another issue to be highlighted was that the study demonstrated the feasibility of measuring the volume of brain structures in clinical magnetic resonance imaging of patients with neurocysticercosis, MTLE, and HS, alone or in different combinations. Cerebral volumetry was performed using a validated, fully automated, and reliable technique that could be easily replicated in future studies.^14^

On the other hand, the present study has some limitations, the most important being the small sample size, which made it impossible to adjust the analyzes for other potentially relevant variables, such as age and head size. Due to the small sample size, the adjustment for multiple tests may have led to a conservative correction with excessive reduction in the ability to detect differences between groups. Other limitations include the fact that MTLE, MTLE‐HS, and neurocysticercosis were defined based on clinical reports and not by a single evaluator.

## 5 CONCLUSION

In our study, we examined whether the analysis of the volume of different brain structures could show changes in patients with neurocysticercosis and/or medial temporal lobe epilepsy with or without hippocampal sclerosis, compared with control patients. After analyzing the data statistically, we found no consistent differences between the study groups.

One of the hypotheses is that our total sample of patients is small and cannot conclude that there is a definite relationship between the volumetric reduction of structures in patients with neurocysticercosis or temporal lobe epilepsy. Another point to be assessed is that there does not seem to be an additive effect of these conditions when combined, versus isolated, in the production of cerebral atrophy.

It is important to note that the feasibility of assessing brain volume in patients with neurocysticercosis and/or MTLE‐HS has been demonstrated, which should lead to further research based on the results of this study, with the possibility of expanding the assessment to other structures and in combination with other pathologies.

## AUTHOR CONTRIBUTIONS

Conceptualization: Jaisa Quedi Araújo, Juliana Ávila Duarte, Marino Muxfeldt Bianchin. Methodology: Jaisa Quedi Araújo, Thiago Junqueira Ribeiro de Rezende, Fabiano Reis, Juliana Ávila Duarte. Investigation: Jaisa Quedi Araújo, Bruna da Silveira Arruda, Sandra Polita, Maurício Anés, Matheus de Lima Ruffini, Adolfo Moraes de Souza, Jordana Soares Chaves. Data curation: Bruna da Silveira Arruda, Matheus de Lima Ruffini. Formal analysis: Jaisa Quedi Araújo, Matheus de Lima Ruffini. Writing—original draft: Jaisa Quedi Araújo, Bruna da Silveira Arruda, Matheus de Lima Ruffini. Writing—review and editing: Adolfo Moraes de Souza, Juliana Ávila Duarte, Marino Muxfeldt Bianchin, Fabiano Reis. Supervision: Juliana Ávila Duarte, Marino Muxfeldt Bianchin.

## FUNDING INFORMATION

This study did not receive any specific grant from funding agencies.

## CONFLICT OF INTEREST STATEMENT

The authors declare no conflicts of interest related to this work.

**Test yourself**

1. What was the main finding regarding brain volumes among the patient groups?

- Significant hippocampal atrophy in NCC patients
- No significant volumetric differences among NCC, MTLE, MTLE‐HS, or combined groups
- Increased limbic system volume in MTLE‐HS patients
- Significant brain volume increase in NCC combined with MTLE‐HS

2 What does the study suggest about the pathophysiological effect of neurocysticercosis (NCC) on brain structure?
  - NCC directly causes significant brain tissue loss and atrophy
  - NCC may act as an initial injury but does not produce measurable volumetric brain changes alone or with MTLE‐HS
  - NCC promotes brain tissue growth and volume increase
  - NCC only affects brain volume during acute infection stages
3 What was concluded about brain volumetric changes in patients with mesial temporal lobe epilepsy (MTLE)?
  - MTLE is associated with significant brain volume reduction regardless of NCC presence
  - MTLE patients show increased brain volumes
  - MTLE combined with NCC causes significant additive brain atrophy
  - MTLE has no impact on brain volume

*Answers may be found in the* *supporting information*.

## Supporting information


Figure S1.

Data S1.

## Data Availability

The data that support the findings of this study are available from the corresponding author upon request.
