# Supplementary material for: Volumetric MRI study of the brain in patients with neurocysticercosis and mesial temporal lobe epilepsy
Source: Epileptic Disord. 2025 Nov 14;28(1):109–18. doi: 10.1002/epd2.70115 (PMC12964183; doi:10.1002/epd2.70115)
Supplement: Supplementary file 1 — Figure S1. [file EPD2-28-109-s001.docx]

**SUPPLEMENTARY MATERIALS**

Supplementary Figure S1. Schematic example of image analysis by Brain GPS^©^
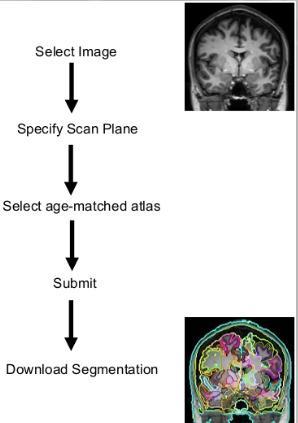


Supplementary Figure S2 – Amygdala volumes across study groups.


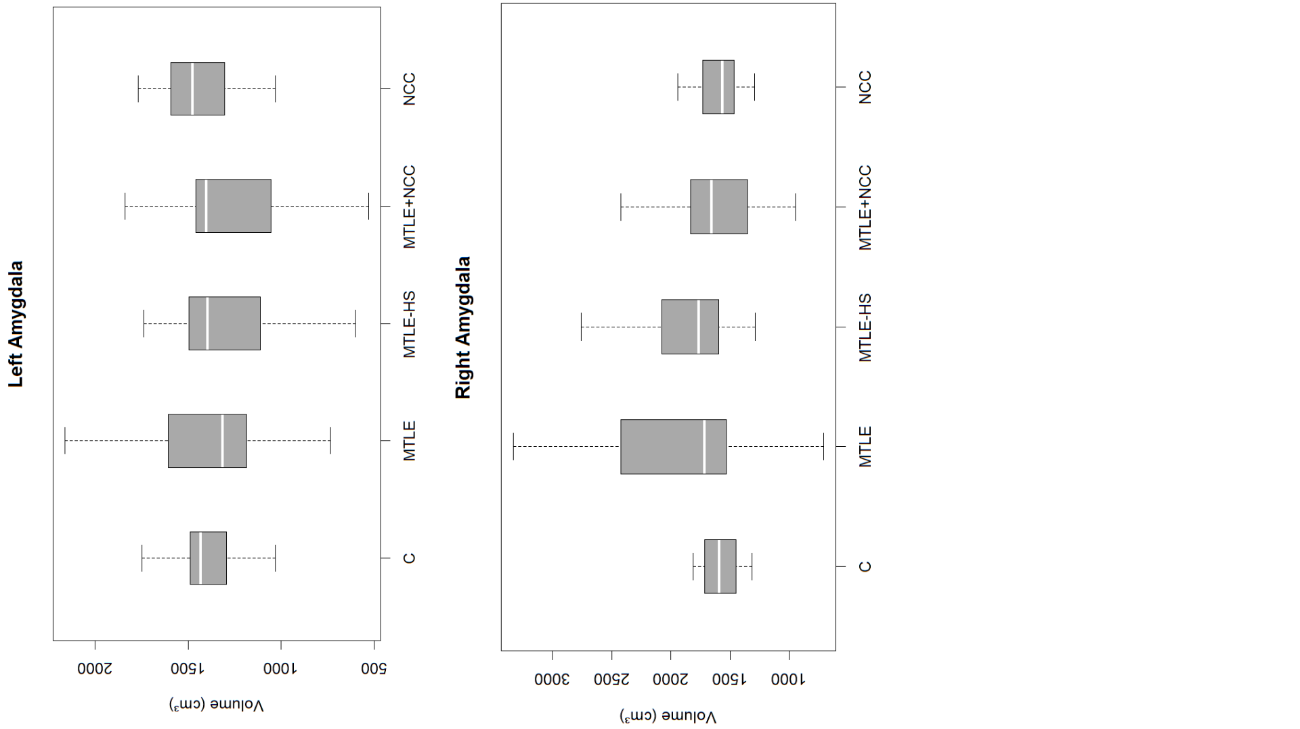


C = Control ; HS = hippocampal sclerosis ; MTLE = mesial temporal lobe epilepsy ; NCC = neurocysticercosis

Supplementary Figure S3 – Cerebellum volumes across study groups.


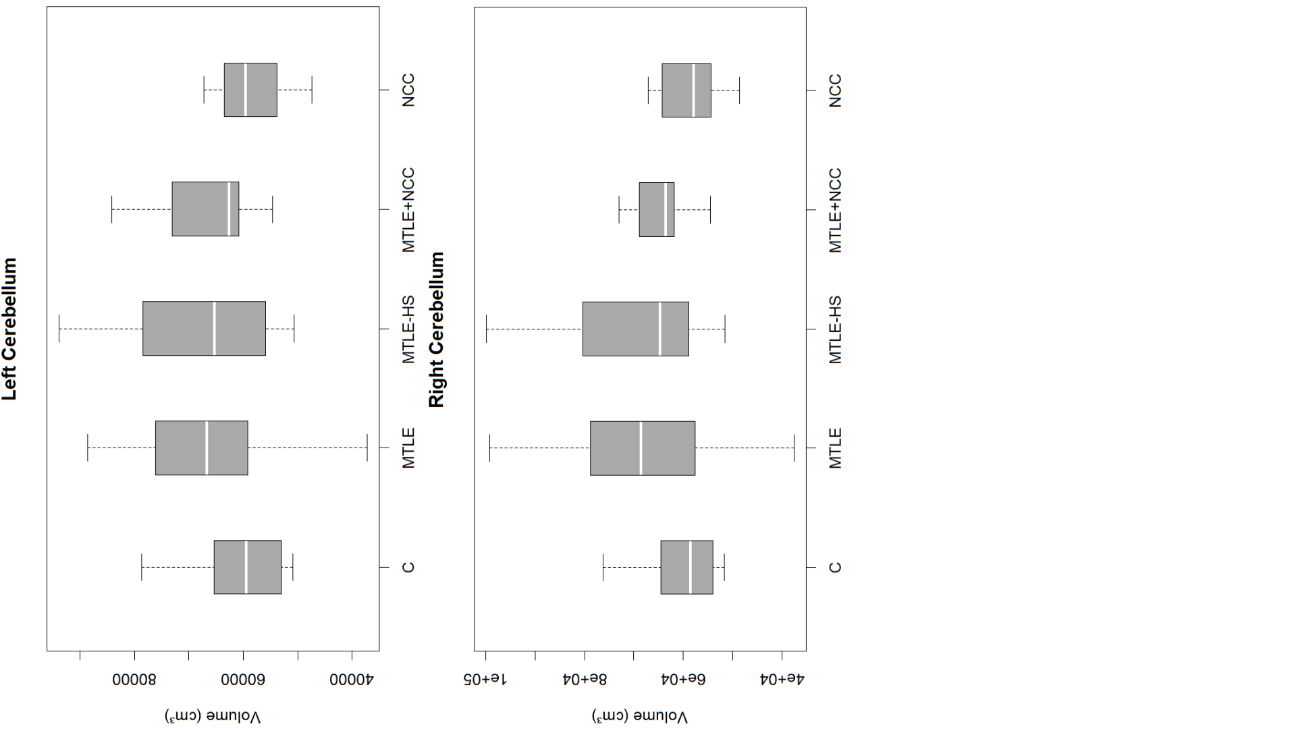


C = Control ; HS = hippocampal sclerosis ; MTLE = mesial temporal lobe epilepsy ; NCC = neurocysticercosis

Supplementary Figure S4 – Frontal Lobe volumes across study groups.


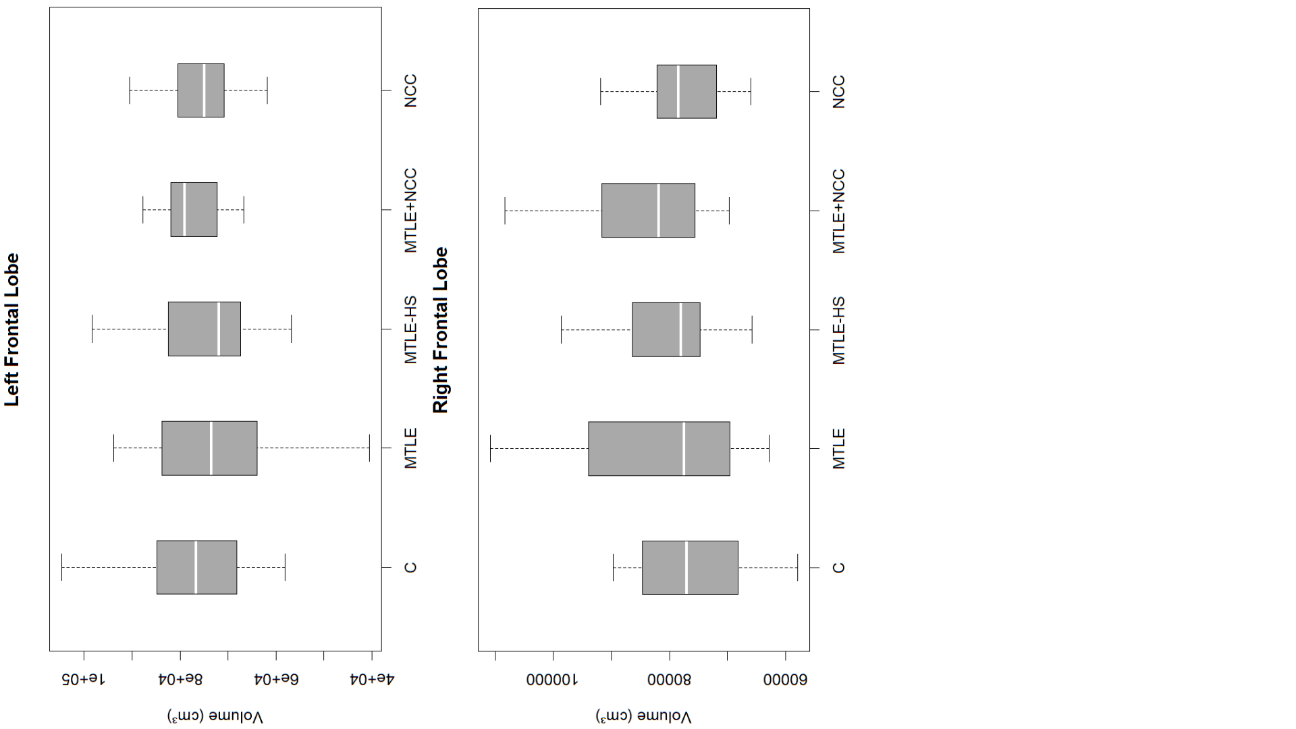


C = Control ; HS = hippocampal sclerosis ; MTLE = mesial temporal lobe epilepsy ; NCC = neurocysticercosis

Supplementary Figure S5 – Limbic System volumes across study groups.


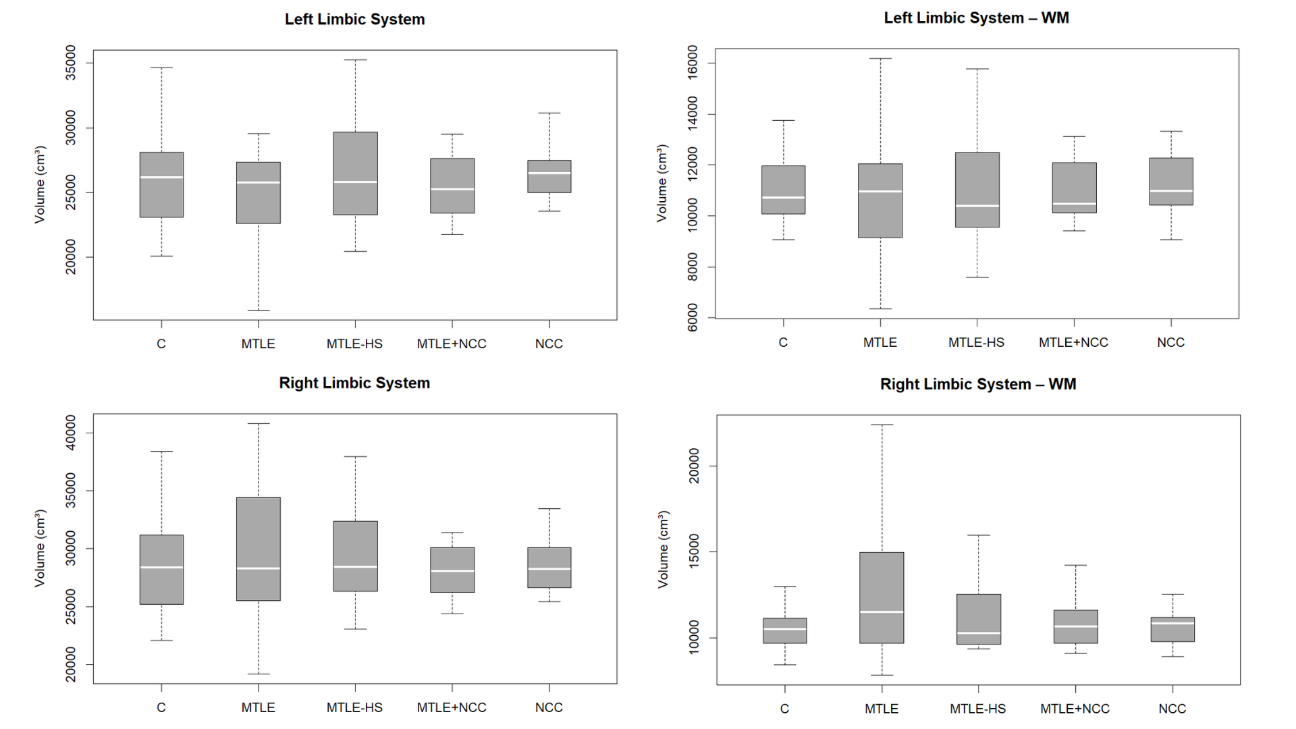
 C = Control ; HS = hippocampal sclerosis ; MTLE = mesial temporal lobe epilepsy ; NCC = neurocysticercosis ; WM = white matter

Supplementary Figure S6 – Occiptal Lobe volumes across study groups.


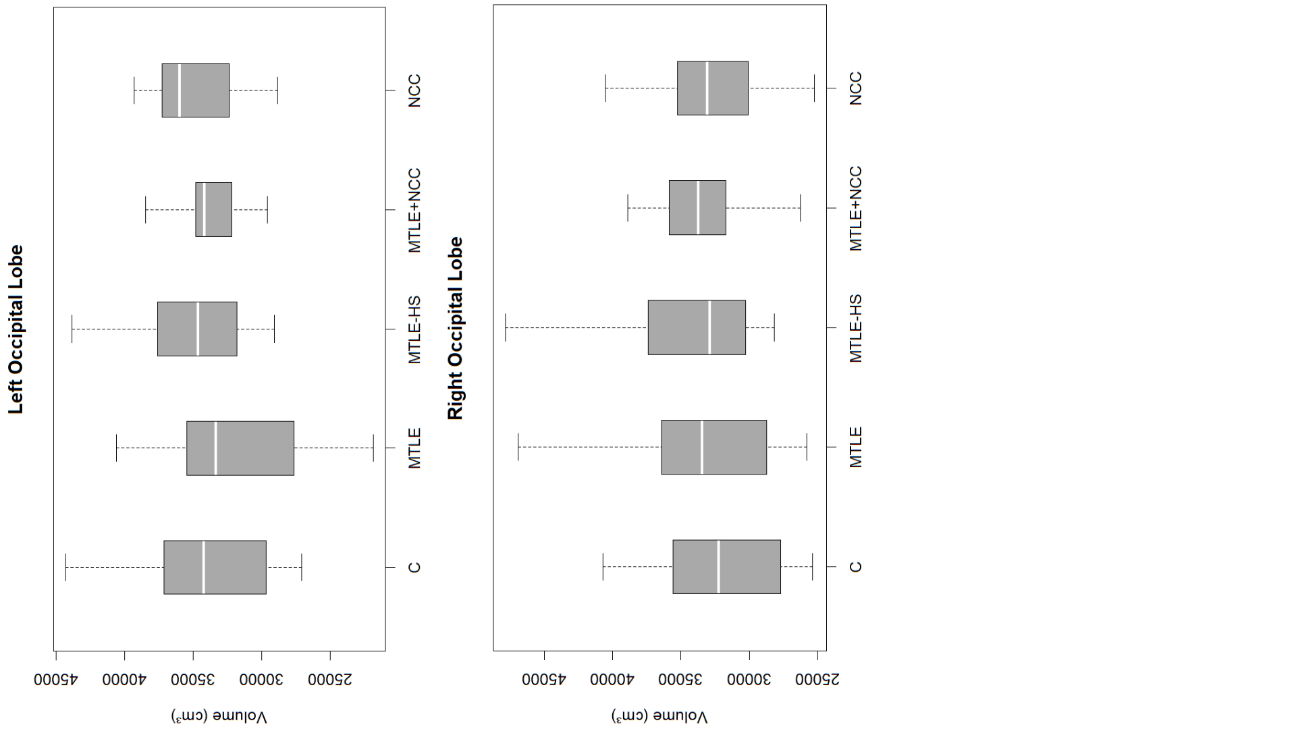


C = Control ; HS = hippocampal sclerosis ; MTLE = mesial temporal lobe epilepsy ; NCC = neurocysticercosis

Supplementary Figure S7 – Parietal Lobe volumes across study groups.


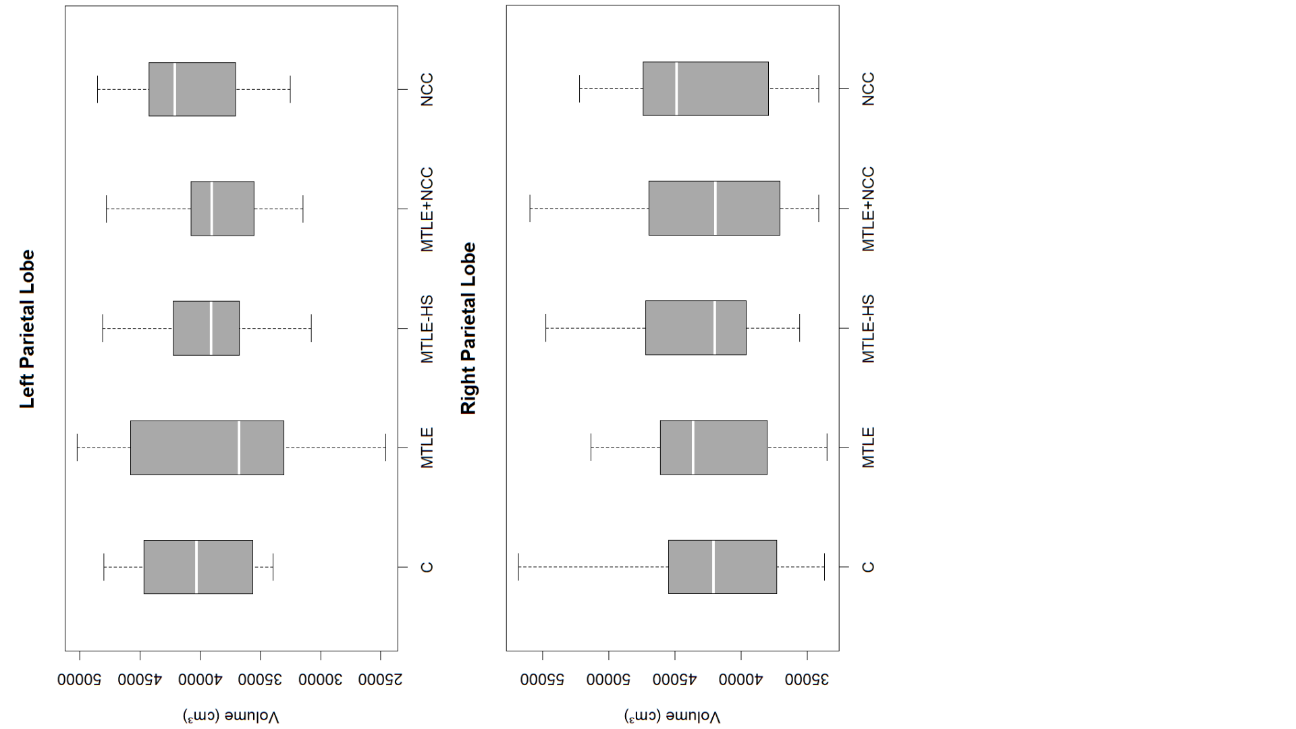


C = Control ; HS = hippocampal sclerosis ; MTLE = mesial temporal lobe epilepsy ; NCC = neurocysticercosis

Supplementary Figure S8 – Thalamus volumes across study groups.


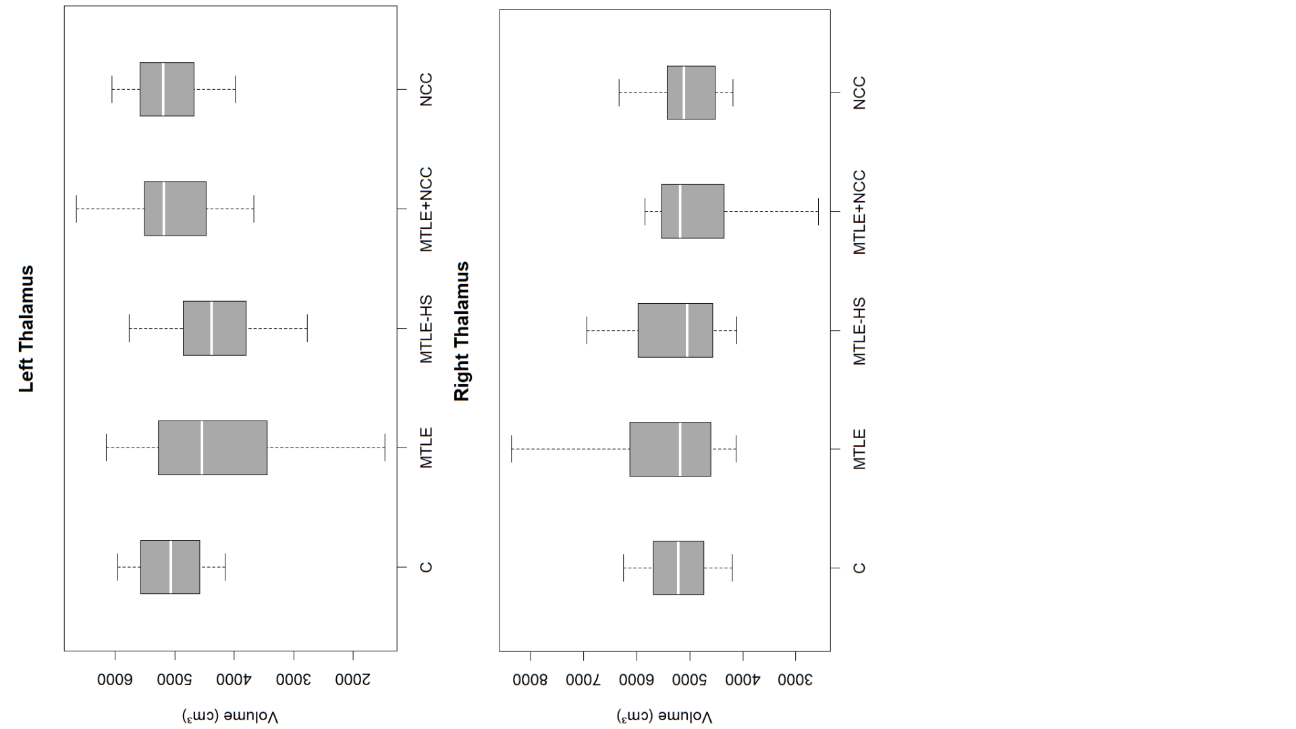


C = Control ; HS = hippocampal sclerosis ; MTLE = mesial temporal lobe epilepsy ; NCC = neurocysticercosis

Supplementary Figure S9 – Total hemispheric and whole cortex volumes across study groups


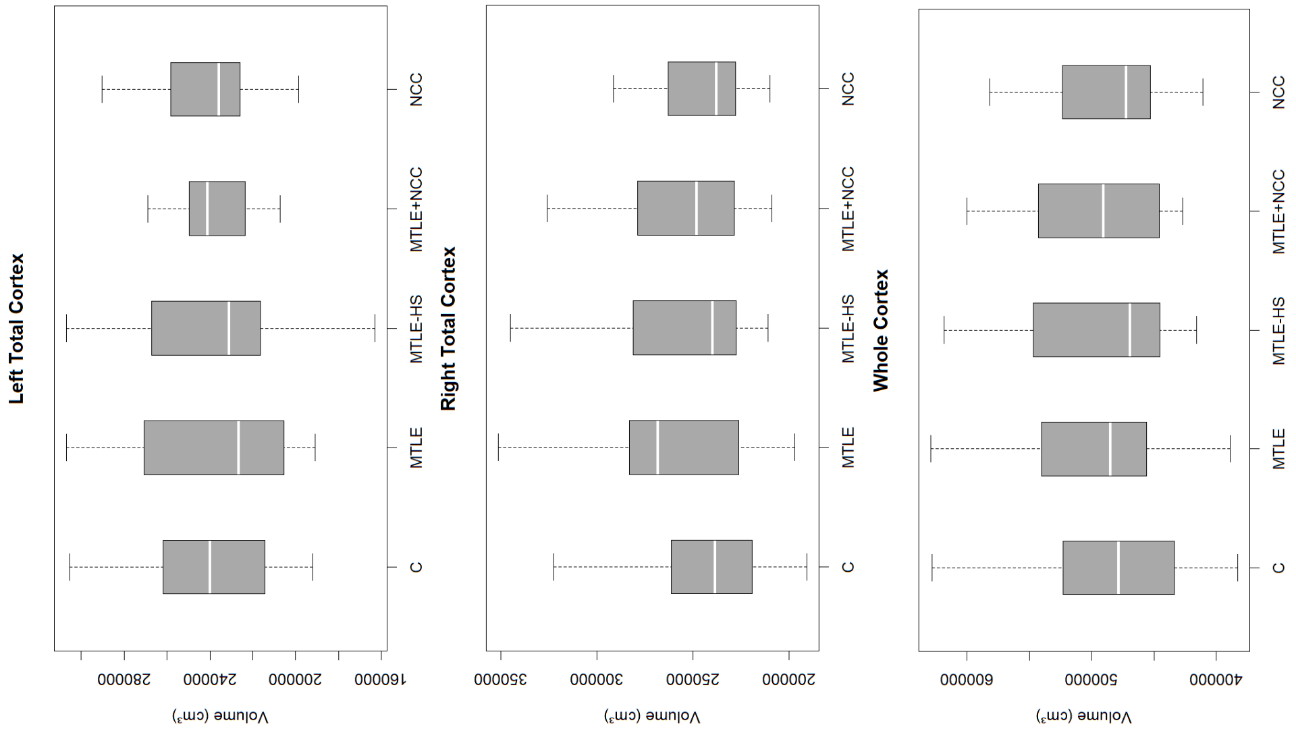


C = Control ; HS = hippocampal sclerosis ; MTLE = mesial temporal lobe epilepsy ; NCC = neurocysticercosis
