# Supplementary material for: Volumetric MRI study of the brain in patients with neurocysticercosis and mesial temporal lobe epilepsy
Source: Epileptic Disord. 2025 Nov 14;28(1):109–18. doi: 10.1002/epd2.70115 (PMC12964183; doi:10.1002/epd2.70115)
Supplement: Supplementary file 2 — Data S1. [file EPD2-28-109-s002.docx]

Test yourself answers

1.

**Answer:** B.

2.

**Answer:** B.

3.

**Answer:** A.
